# “Omic” investigations of protozoa and worms for a deeper understanding of the human gut “parasitome”

**DOI:** 10.1371/journal.pntd.0005916

**Published:** 2017-11-02

**Authors:** Valeria Marzano, Livia Mancinelli, Giorgia Bracaglia, Federica Del Chierico, Pamela Vernocchi, Francesco Di Girolamo, Stefano Garrone, Hyppolite Tchidjou Kuekou, Patrizia D’Argenio, Bruno Dallapiccola, Andrea Urbani, Lorenza Putignani

**Affiliations:** 1 Human Microbiome Unit, Bambino Gesù Children’s Hospital IRCCS, Rome, Italy; 2 Laboratory Medicine, Bambino Gesù Children’s Hospital IRCCS, Rome, Italy; 3 Pediatric Immuno-infectivology, Bambino Gesù Children's Hospital IRCCS, Rome, Italy; 4 Scientific Directorate, Bambino Gesù Children's Hospital IRCCS, Rome, Italy; 5 Institute of Biochemistry and Biochemical Clinic, Faculty of Medicine and Surgery–Policlinico A. Gemelli, Catholic University of Sacred Heart, Rome, Italy; 6 Proteomic and Metabonomic Unit, Fondazione Santa Lucia IRCCS, Rome, Italy; 7 Parasitology Unit, Bambino Gesù Children’s Hospital IRCCS, Rome, Italy; James Cook University, AUSTRALIA

## Abstract

The human gut has been continuously exposed to a broad spectrum of intestinal organisms, including viruses, bacteria, fungi, and parasites (protozoa and worms), over millions of years of coevolution, and plays a central role in human health. The modern lifestyles of Western countries, such as the adoption of highly hygienic habits, the extensive use of antimicrobial drugs, and increasing globalisation, have dramatically altered the composition of the gut milieu, especially in terms of its eukaryotic “citizens.” In the past few decades, numerous studies have highlighted the composition and role of human intestinal bacteria in physiological and pathological conditions, while few investigations exist on gut parasites and particularly on their coexistence and interaction with the intestinal microbiota. Studies of the gut “parasitome” through “omic” technologies, such as (meta)genomics, transcriptomics, proteomics, and metabolomics, are herein reviewed to better understand their role in the relationships between intestinal parasites, host, and resident prokaryotes, whether pathogens or commensals. Systems biology–based profiles of the gut “parasitome” under physiological and severe disease conditions can indeed contribute to the control of infectious diseases and offer a new perspective of omics-assisted tropical medicine.

## Introduction

Every human subject has a specific gut microbiota that may change over their life span due to complex interactions between host genetics, immune response, diet, and environment [1] under physiological and pathological conditions [2]. Indeed, several recent studies have demonstrated the multitude of ways by which the microbiota has influenced human health and physiology [3]. Alterations in the human gut microbiota have been associated with a range of illnesses in the developed world, including inflammatory bowel disease (IBD), obesity, type 2 diabetes, allergies, and even autism, through the gut–brain axis [4, 5].

The definition of microbiota is related to the complex community of microorganisms mainly composed of bacteria but also including viruses, Archaea, eukaryotes such as fungi, and protozoa living in consortia in sites such as the gastrointestinal (GI) tract [3]. The human gut “virome,” composed mainly of bacteriophages [6], and the “mycobiome,” composed of yeasts and other fungi [7], have the potential to modify and regulate bacterial communities and hence modify and regulate human health. Among others, parasitic protozoa [8, 9], worms [10], and even eukaryotic commensals, such as *Blastocystis hominis* and *Dientamoeba fragilis* [11], can be of great importance. Particularly, it is still a matter of debate whether *Blastocystis* is associated or not with gut dysbiosis conditions [12], while interactions between helminths, protozoans, and the host immune system have been demonstrated [13, 14], as in the case of intestinal helminths, whose absence in the gut has been proposed as a risk factor for allergic/autoimmune/inflammatory diseases, including IBD [13, 15, 16]. Moreover, interactions between parasites and bacterial communities in the human gut may have a profound impact on the alteration of parasite virulence, course of both mucosal and systemic parasitic infection, and host immune response to the parasite, possibly explaining the observed variability in disease expression [14, 17, 18].

Based on such considerations, it is foreseeable that the exploration of parasites, protozoa, and worms within microbiota communities by “omic” technologies may provide more fully comprehensive information on gut prokaryote profiles.

**Fig 1:**
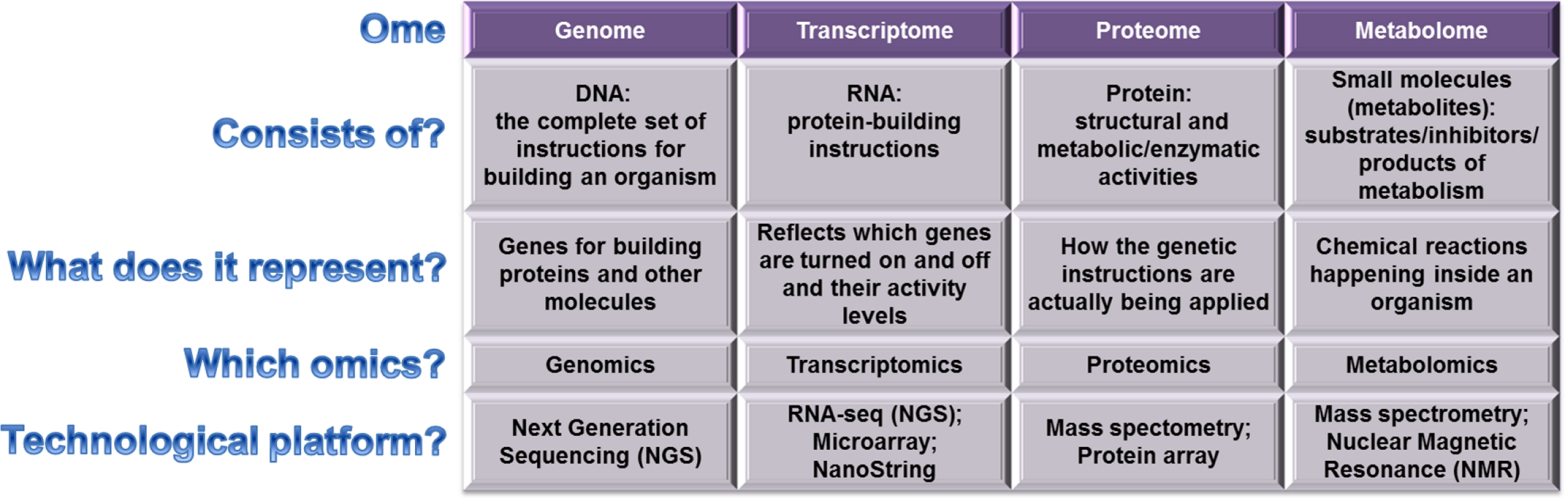
Summary of current “omic” technologies for “parasitome” investigations.

Such “omics”-based approaches are built on a holistic vision of the systems analysed, systems in which “all components are considered in complex ecological networks” in order to provide complete profiles of genes/transcripts/proteins/metabolites (Fig 1) [19, 20].

When applied to the study of prokaryotic consortia/communities, these technologies are denominated metagenomics, metatranscriptomics, metaproteomics, and meta-metabolomics and allow a non-targeted and high-throughput searching of the genetic scaffold and functional reservoir of the microbiota system, providing profiles of different organisms at the same time [21, 22]. Although some findings from metagenomic and metabolomic approaches to neglected tropical diseases are already available [23], meta-omic developments in the study of human gut “parasitome” are still in their infancy.

In this review, “omic” technologies (Table 1), applied to the study of human gut parasites (i.e., gut “parasitome”), are presented with the aim of furthering the understanding of gut parasite impact on intestinal ecology and dysbiosis. Much relevant work has been done on “model” parasites that are not primarily associated with the gut—e.g., liver flukes and blood flukes. As an example, an integrated transcriptomic and proteomic approach allowed researchers to describe the *Fasciola hepatica* secretory proteome, thus identifying proteins such as cathepsin, peroxiredoxin, glutathione S-transferase (GST), and fatty acid-binding proteins (FABPs) essential for the design of the first-generation anti-fluke vaccines and flukicidal drugs [24]. FABPs were also found to be the most abundant excretion/secretion proteins (ESPs) of *Schistosoma japonicum*, highlighting the vital importance of these proteins in the evasion process from the host immune system [25]. Based on these pivotal discoveries, *Schistosoma* FABPs and GSTs were selected by the World Health Organization as anti-*Schistosoma* vaccine candidates [26].

**Table 1:** Scheme of reported investigations on a human or animal model gut “parasitome” by exploiting “omic” technologies.

Parasite	“Omic” approach	Sample	Technique	Major results	Reference
*Ancylostoma caninum*	Proteomics	Adult worm from small intestines of stray dogs	1-DE LC-ESI-MS/MS^a^; protein or peptide OGE^b^ and shotgun LC-ESI-MS/MS	Description of proteins from the excretory/secretory products	[74]
*Ancylostoma ceylanicum*	Genomics	Hookworms culture and infection in golden hamster (*Mesocricetus auratus*)	Genomic library; Illumina sequencing	Complete sequence of hookworm genome and definition of temporal genes expression	[48]
	Transcriptomics		RNA-seq library, Illumina sequencing		
*Blastocystis* spp.	Metagenomics	Faecal DNA from 2 groups of patients, positive or negative for *Blastocystis*	NGS^c^: Ion Torrent sequencing	*Blastocystis* colonisation is positively associated with increased bacterial diversity and with higher abundance of Clostridia class, Ruminococcaceae, and Prevotellaceae	[37]
		Faecal DNA from cohorts of healthy individuals and patients with Crohn’s disease, ulcerative colitis	NGS: WGS^d^ Illumina sequencing	*Blastocystis* colonisation is positively associated with species richness and with species present in lean individuals, such as the *Prevotella* or *Ruminococcus* enterotype	[33]
*Blastocystis* ST7	Proteomics	Axenic culture of an isolate obtained from a Singaporean patient with intestinal disorders	1-DE LC-ESI-MS/MS^a^	Definition of secreted protease candidates for the effects induced at the parasite–host interface and involved in mucus degradation: legumain peptidase ACY95293.1, and cathepsin B peptidase CBK25506.2.	[72]
*Cryptosporidium parvum*	Proteomics	Excysted sporozoites from lambs	2-DE MALDI TOF^e^; 1-DE LC-ESI-MS/MS; Shotgun MudPit^f^ LC-ESI-MS/MS	Definition of sporozoite proteome	[59]
		Excysted and non-excysted calf oocysts	Stable isotope labelling iTRAQ^g^ LC-ESI-MS/MS; Shotgun LC-ESI-MS/MS	Definition of excysted and non-excysted oocysts proteome; specific proteins augmented in excysted invasive sporozoites: many ribosomal (40S and 60S) and heat shock chaperonin (Hsp70 and Hsp90)	[60]
	Metabolomics	Human faecal samples	Untargeted GC-MS^h^	Higher abundance of phenylalanine, valine, isoleucine, serine, succinic acid, and threitol, lower levels of xylose in infected sample; faecal metabolite profiles generated are able to identify infected individuals	[86]
		Faecal sample from mice infected with S26 isolate (obtained from a naturally infected calf)	Chemical Derivatization GC-MS	Less abundant metabolites and intermediaries involved in energy metabolism (key nutrients scavenged by *Cryptosporidium* to supplement its metabolic pathway) were detected in infected mice than in uninfected mice	[87]
*Encephalitozoon cuniculi*	Proteomics	GB-M1 cultured in Madin-Darby canine kidney or human foreskin fibroblast cells	2-DE MALDI-TOF/ESI LC-MS/MS; Shotgun LC-ESI-MS/MS	Identification of a reference map of the major proteins expressed during late sporogony	[62]
*Entamoeba histolytica*	Proteomics	Axenic cultured trophozoites (strain HMI:IMSS)	2-DE LC-ESI-MS/MS	Identification of specific parasite proteins that promote host invasion	[68]
			1-DE LC-ESI-MS/MS	Identification of the cell surface–associated proteome	[67]
		Axenic cultured trophozoites (strains HMI:IMSS and Rahman)	2-DIGE^i^ MALDI-TOF/ESI LC-MS/MS	Identification of important molecular component defining physiologically relevant virulence phenotype	[70]
*Entamoeba histolytica* and *Entamoeba dispar*	Proteomics	Axenic cultured trophozoites (strain HMI:IMSS of *E*. *hystolytica* and SAW760 of *E*. *dispar*)	2-DE MALDI-TOF	Identification of more proteins which are involved in *Entamoeba* pathogenicity	[69]
*Entamoeba invadens*	Transcriptomics	Axenic cultured trophozoites (IP-1 strain)	Affymetrix platform microarray	First description of transcriptional and metabolic regulatory networks dynamics taking place during *E*. *invadens* encystation	[89]
	Metabolomics		CE^j^-ESI-TOF-MS/MS		
*Giardia duodenalis*	Metabolomics	Stool from patients with confirmed infection and controls with diarrhoea	GC-MS	First report of the Volatile Organic Compounds that could be biomarkers of *Giardia* infection	[88]
*Giardia lamblia*	Proteomics	Cultured WBC6 (ATCC catalog number 50803) trophozoites	1-DE LC ESI-MS/MS	Definition of the protein repertoire of peripheral and encystation-specific vesicles that have key roles in proliferation and transmission to a new host	[61]
*Heligmosomoides polygyrus*	Metagenomics	Distal ileum and cecal tip of C57BL/6 healthy and infected mice; L3 larvae and adult worms from duodenum of infected mice	NGS: 16S rRNA gene targeted amplification and Sanger-style sequencing; quantitative PCR clone library analysis	Infection significantly alters the gut microbiota with increased Lactobacillaceae bacterial load	[42]
Helminths (*Trichuris* spp., *Ascaris* spp., hookworm)	Metagenomics	Faecal sample of helminth-infected or uninfected indigenous Malaysians and New York City residents	NGS: V4 16S rRNA gene targeted amplification; Illumina sequencing	Significant effect of helminth colonisation on the diversity, bacterial community structure and function of the gut microbiota	[45]
*Necator americanus*	Metagenomics	Faecal samples of experimentally infected human volunteers (affected by celiac disease) on a gluten-free diet	NGS: 16S rRNA gene targeted amplification; 454 pyrosequencing	Hookworm infection did not have a major impact on the community structure of the intestinal microbiota	[41]
		Prior and post dietary gluten exposure faecal samples of experimentally infected human patients (affected by celiac disease)		Microbial species richness increases during the challenge with escalating doses of dietary gluten, a potential mechanism by which hookworm infection could positively impact gluten-induced inflammation and intestinal immune homeostasis	[40]
	Genomics	L3i and adult worms from intestines of Golden Syrian Hamster infected subcutaneously with the Anhui strain	NGS: WGS and 454 pyrosequencing	Draft genome sequence and postgenomic analyses to unveil the immunobiology of human hookworm disease	[49]
	Transcriptomics		Rna-seq: Roche/454 and Illumina cDNA libraries; Genome Sequencer Titanium FLX and Illumina sequencing		
	Proteomics		OGE and shotgun LC-ESI-MS/MS; protein microarray		
	Metabolomics	Urine and blood samples from infected and control Syrian hamsters	^1^H NMR^k^	Unveil the biochemical consequences of infection	[90]
		Urine and blood samples from infected and control Syrian hamsters coinfected with *Schistosoma japonicum*			[91]
*Strongyloides stercoralis*	Transcriptomics	Parasites from faecal samples of infected individual of the endemic area of La Safor (Valencia, Spain) and propagated on axenic culture	cDNA library, 454 pyrosequencing	First comprehensive database of third larval stage transcripts	[65]
	Proteomics		Shotgun LC-ESI-MS/MS	First study of the *S*. *stercoralis* proteome	[64]
*Taenia solium*	Proteomics	Gravid proglottids from Peruvian patients' stools	LC fractionation and MALDI TOF MS/MS	Definition of oncosphere proteome	[63]
		Metacestodes from naturally infected pigs in Zambia and Perú	1-DE LC-ESI-MS/MS	First report of the metacestode excretion/secretion proteome	[73]
*Trichinella spiralis*	Proteomics	Worms (isolated from the small intestine of infected rats) cocultured with different strains of bacteria	iTRAQ bidimensional LC-ESI-MS/MS	Comprehension of microbe-induced alterations in the survival and reproduction of *T*. *spiralis* in vitro: *Lactobacillus bulgaricus* and *L*. *acidophilus* were beneficial; *Salmonella enterica* and *Escherichia coli* O157:H7 (EHEC) were not	[76]
*Trichuris suis*	Metagenomics	Luminal colon content of control and infected piglets	NGS: 16S rRNA gene targeted amplification and WGS 454 pyrosequencing	Identification of the infection significant impact on the proximal colon microbiota composition at both the phylum and genus levels (key genera: *Mucispirillum*, *Succinivibrio*, and *Ruminococcus*) supported by metabolic and functional data	[43]
	Metabolomics		GC-MS		
*Trichuris trichiura*	Genomics	A clinically isolated adult male	NGS: WGS, Illumina sequencing	High-quality draft genome assembly	[79]
	Transcriptomics	Stool sample of infected Ecuadorian children	NGS: Ion Torrent sequencing	First transcriptomic analysis of the adult stage of the human whipworm	[78]
	Proteomics		Shotgun LC-ESI-MS/MS	Identification of proteins with immunomodulatory effects	[77]
Unknown parasites	Metagenomics	Wild rat faeces	NGS: V9 18S rRNA gene targeted amplification; Illumina sequencing	Novel method to determine host alimentary tract parasite infections	[50] [51]

^a^ 1-DE LC-ESI-MS/MS: monoDimensional gel Electrophoresis Liquid Chromatography-ElectropSprayIonisation-tandem Mass spectrometry

^b^ OGE: OFFGEL fractionation by isoelectric focusing

^c^ NGS: Next Generation Sequencing

^d^ WGS: Whole Genome Sequencing

^e^ 2-DE MALDI TOF: biDimensional Matrix-Assisted Laser Desorption/ionization Time-of flight mass spectrometry

^f^ MudPit: Multi-dimensional Protein Identification technology

^g^ iTRAQ: isobaric Tags for Relative and Absolute Quantitation

^h^ GC-MS: gas chromatography-mass spectrometry

^i^ 2-DIGE: bidimensional-DIfference Gel Electrophoresis

^j^ CE: Capillary electrophoresis

^k 1^H NMR: Proton Nuclear Magnetic Resonance spectroscopy

ESP-induced early changes in host cells highlighted by proteomics were also confirmed for *Opisthorchis viverrini* [27, 28], and indeed, plasma actin-related protein 3 (ARP3) autoantibody and 14-3-3 eta protein were identified as putative new diagnostic markers of opisthorchiasis [29, 30].

Below, “omics”-based investigations on human gut protozoa and worms will be discussed to update the state of the art on gut “parasitome” citizens by “omic” technologies.

## Methods

Literature searches in PubMed until October 31, 2016 were performed using a search strategy designed to identify relevant studies for this review from the following 2 categories: (i) evaluating genes, transcripts, proteins, and metabolites of parasites colonising/invading the human gut and (ii) approaches in “omics”-based research methodologies. Two investigators independently evaluated articles resulting from these searches and any relevant references cited in those articles for inclusion in this study.

## DNA-based “omics”: Genomics and metagenomics

Since the beginning of the 21st century, tremendous advances in DNA sequencing technologies have emerged, allowing for the study of genomes in greater depth and therefore better decoding of their structural and functional attributes. In particular, next-generation sequencing (NGS) technologies have displayed high-throughput sequencing power and are composed of a number of different modern methods that sequence nucleotides faster and cheaper than Sanger capillary electrophoresis, including HiSeq/MiSeq Illumina, Roche 454, Ion torrent: Proton/PGM, SOLiD, PacBio, and Oxfordnanopore platforms [31].

Briefly, NGS pipelines are based on (a) sample collection, (b) nucleic acid extraction, (c) library and (d) template preparation, and (e) sequencing reaction completed by (f) genome and read alignments during the data analysis [32]. The application of NGS could be basically divided into the following 2 categories: (a) de novo genomic sequencing by mate-paired and whole genome shotgun (WGS) strategies for determining the complete DNA sequence of an organism’s genome at a single time and (b) targeted sequencing of gene or locus for the analysis of specific mutations and phylogenetic and evolutionary studies. 16S rRNA for bacterial and 18S rRNA for eukaryotic genes are the most investigated targeted sequences because their high degree of sequence conservation across many groups of organisms provides the most suitable method for microorganism identification by so-called targeted metagenomics.

### Metagenomics to highlight gut bacterial and eukaryotic relationships

NGS platforms allow metagenomic studies, which have revolutionised microbiology and related fields, to investigate whole prokaryotic communities in terms of the presence and relative abundance of microorganisms. Metagenomics is potentially important in describing the interplay of prokaryotic communities with their eukaryotic counterparts within the whole intestinal ecological system. By using this approach, *Blastocystis* was described in healthy individuals but not in patients with Crohn’s disease [33]. Particularly, lower *Blastocystis* colonisation levels were observed in subjects characterised by a *Bacteroides*-driven enterotype as compared with *Prevotella*- or *Ruminococcus*-driven enterotypes and were positively associated with species richness [33]. This aspect is particularly interesting because disease enterophenotypes, such as those related to IBD, obesity, and nonalcoholic fatty liver disease/steatohepatitis (NAFLD), are generally inversely related to bacterial richness [34–36]. Hence, *Blastocystis* may be considered a richness-dependent marker [33, 37].

Clearly, the biocomplexity of the intestinal lumen suggests that interactions between parasites and the intestinal microbiota would also influence inflammation. Recent studies have investigated the potential therapeutic properties of GI nematodes in modulating regulatory responses in the host gut and thereby promoting immune homeostasis [38]. Epidemiologic studies noted a reduced susceptibility to inflammatory diseases (e.g., asthma) in the presence of hookworm infection [39]. Indeed, it has been suggested that *Necator americanus* may alleviate chronic inflammation in celiac disease but also maintain prokaryotic species richness, thereby reestablishing the GI tract’s microbial eubiosis and immune homeostasis [38, 40]. Probably, the effects of *N*. *americanus* is explicit on microbiota species richness rather than on community structure or relative abundance of individual bacterial species [41].

In an interleukin-10 (IL-10) gene-deficient murine model of IBD, infection by *Heligmosomoides polygyrus* was evaluated for treatment of colitis, and indeed, the amelioration of colonic inflammation was observed in wild-type C57BL/6 mice [42]. One proposed mechanism was that *H*. *polygyrus* infection favours the outgrowth or suppression of certain bacteria, which in turn help modulate host immunity. Indeed, Lactobacillaceae significantly increased in abundance in the ileum of the infected mice, supporting the concept that helminth infection shifts the composition of intestinal bacteria [42]. The alteration of prokaryotic community structure was also observed in an animal model (pig) during a *Trichuris suis* infection [43]. Interestingly, the meta-taxonomy alterations were detected by both targeted metagenomics and WGS sequencing. Amongst the 15 phyla identified, the abundances of Proteobacteria and Deferribacteres were changed in infected pigs (IPs). Seventeen genera, such as *Oscillibacter*, *Succinivibrio*, *Sporobacter*, *Spirochaeta*, *Paraprevotella*, and *Mitsuokella*, were significantly affected (*P* ≤ 0.05). The relative abundance of *Oscillibacter*, the second most abundant genus in the colon microbiota of control pigs (CPs), decreased from 7.8% of CPs to 2.8% of IPs. Similarly, the relative abundance of *Succinivibrio* decreased from 3.6% of CPs to 0.4% of IPs. On the other hand, an 86-fold expansion in the relative abundance of *Mucispirillum* to 0.09% was registered in IPs, accounting for all observed changes in the phylum Deferribacteres [43].

Because “parasitome” is strictly related to environmental (e.g., geographic and temporal clusters, etc.) and host determinants of parasite infection (e.g., age, immunological status, travels, community behaviours) [44], socioeconomic and anthropologic factors were also evaluated in a study on helminth colonisation and alterations of gut microbiota in a group of Malaysian indigenous people [45]. An increased ecological diversity and a higher abundance in the classes Alphaproteobacteria and Mollicutes, the order Bacteroidales, and in particular, its family Paraprevotellacae were observed in helminth-infected people. In contrast, helminth-negative people showed an increased abundance in the *Bifidobacterium* spp. [45]. Although differences in the distribution of bacterial operational taxonomic units (OTUs) between infected and noninfected Malaysian people were smaller than those observed between urban United States and Malaysian residents, higher bacterial diversity (i.e., α- and β-diversity) definitely appeared associated with helminth colonisation, once more suggesting that helminth-driven alterations of microbiota are especially evident in terms of richness [45].

On the other hand, *Trichuris*-driven infections were observed to ameliorate colitis by restoring mucosal barrier functions acting on mucus production and on diversity of mucosal bacteria in a macaque model [46].

The above diversity in metagenomic reports are clearly due to different helminth species/hosts investigated, dissimilar infectious doses used to inoculate human volunteers or animal models, different technological platforms, and the different sample types studied (e.g., mucosa versus faeces); however, such diversity of conclusions is also linked to the high complexity of gut prokaryotic and eukaryotic communities. Hence, the standardisation of “omic” procedures can represent the first step to making more homogenous report inferences.

### Future of DNA-based studies on “parasitome” investigations: The challenge of sequencing parasite genomes

The standardisation of procedures and a comparison of different results from different metagenomic pipelines are completely lacking in the specific field of “parasitome” characterisation. High sequence similarities between related species and/or the absence of parasite sequences in available current databases are still the major weaknesses of metagenomics-driven approaches to obtaining accurate species-level identification of parasites. Enormous efforts have been undertaken during the past few years to increase the availability of extended parasite databases (http://parasite.wormbase.org/, http://www.sanger.ac.uk/science/collaboration/50HGP, http://eupathdb.org/eupathdb/ [47]), as in the case of *Ancylostoma ceylanicum* [48] and *N*. *americanus* [49], for example. Hopefully, in the near future, genomes of more parasites will become available. Nevertheless, 18S rDNA-based metagenomic approaches developed to facilitate the detection of eukaryotic parasites are characterised by a sensitivity at least as high as the conventional microscopy-based method [50, 51].

### Epigenomics to highlight gut “parasitome” and host relationships

Among the application of NGS technologies, there is the study of the epigenome, i.e., the study of any potentially stable and heritable changes in gene activity and expression without altering DNA sequences. DNA methylation and histone modifications are examples of tightly regulated mechanisms that produce such DNA changes.

DNA methylation sequencing and chromatin immunoprecipitation followed by sequencing (ChIP -Seq) enable the precise genomic localisation of epigenetic markers to decipher gene activity and expression as well as chromatin state. There is growing interest in epigenetics for its role in the development and reproduction of parasites and host–parasite interactions through potentially mutual modulation of genomes. Epigenetic studies of parasites are mostly linked to malaria and schistosomiasis; only a few are related to gut-related parasitoses. Amongst protozoa, *Cryptosporidium parvum *encodes candidate methyltransferases, although no proteins were identified; in *Entamoeba histolytica* and *Trichinella spiralis*, methylated DNA was identified, as well as the presence of DNA methyltransferases or their coding genes [52]. Also, the modulation of epigenetic host processes was demonstrated in helminth-induced immune suppression [53]. Future epigenetic research on parasites will provide us with better knowledge of both environmental signals and parasite sensor and executor molecules, which determine different parasite development and virulence programs. Moreover, this knowledge will give us potential opportunities for disease intervention.

## RNA- and protein-based “omics”

The identification and quantification of transcriptionally active regions of the genome (the transcriptome) and of the ultimate products of the transcription (the proteome) are of fundamental importance to elucidate biological functions. NGS technologies allow us to also examine all the RNA and its differential expression, thanks to an additional step of the operative pipeline: after RNA extraction, researchers synthesise complementary DNA (cDNA) from RNA [54]. Proteomics deals with the determination of proteins of a biological system by biophysical and biochemical methods. It has been revolutionised by the advent of mass spectrometry (MS), a sensitive and rapid analytical method for protein identification and quantification that enables large-scale, fast, and systematic measurements of proteomes in space and time. Basically, an MS proteomic experiment consists of the following: (a) protein extraction and purification from matrices, (b) direct analysis (top-down approach) or enzymatic digestion (bottom-up approach), (c) optional protein/peptide separation based on liquid chromatography, (d) mass-to-charge and intensity detection of protein/peptide and their induced fragments by MS, and (e) protein identification and quantification by de novo or database-driven data analysis [55].

Gut parasites have been analysed by transcriptomics or proteomics to highlight invasive and diagnostic features, while, to our knowledge, metatranscriptomic or metaproteomic studies of gut prokaryotic and eukaryotic communities, including parasites, have not yet been performed.

### Elucidation of parasite life cycle stage–specific characteristics

Transcriptomic and proteomic studies may provide evidence of a parasite’s gene expression products in order to have a more comprehensive, functional picture of each parasite’s vital stages and metabolism [22, 56]. This effort has been supported by the introduction of advanced bioinformatic resources for the handling of transcriptomic and proteomic data, which integrate whole genome sequences and annotations with expressed sequence tags [47, 57, 58]. So far, proteomics has described the following parasite cycle features: (i) secretion and excystation proteins of *C*. *parvum* oocyst/sporozoite stages [59, 60]; (ii) *Giardia* trophozoites’ peripheral and encystation-specific vesicles, organelles that have key roles in proliferation and transmission to a new host, respectively [61]; (iii) *Encephalitozoon cuniculi* spore-rich cell populations, representing the major protein reservoir expressed during late sporogony [62]; (iv) *Taenia solium* activated oncospheres, involved in gut penetration and immune evasion machineries [63]; and (v) *Strongyloides stercoralis* infective filariform larvae (L3i) [64]. Moreover, the transcriptome of *S*. *stercoralis* L3i has been annotated to provide a comprehensive database for genomic, proteomic, and metabolomic explorations of *S*. *stercoralis* [65].

### Focus on parasite surface and secreted proteins

Several proteomic investigations have focused on surface proteins because they are thought to be involved in the host–parasite interactions, immune response, and disease processes [66]. In the case of *E*. *histolytica*, more proteins than expected have been recently catalogued as surface associated, a phenomenon that may be caused by its high membrane turnover [67]. The surface-associated proteome can indeed elucidate molecular mechanisms that regulate virulence in *E*. *histolytica* [68]. A large number of proteins that can potentially act as new virulence factors were highlighted by comparing the proteome of *E*. *histolytica* with that of the closely related but nonpathogenic *E*. *dispar* [69] and investigating different virulent *E*. *histolytica* strains [70].

Gut parasite secretory products have been intensively studied, especially with reference to their proteolytic activities. Several enteric pathogens, in fact, can modulate the protease balance, which regulates the intestinal epithelial cell microenvironment, inducing intestinal pathobiology [71]. Moreover, specific parasite-secreted proteases stimulate gut secretion or inflammation, alter gut integrity, inhibit host immunity, and therefore promote disease, such as in *Entamoeba* spp. or *Giardia* spp. infections, in which cysteine proteases are effective virulence factors. Secreted proteases were also characterised in *Blastocystis* culture supernatants, allowing protease candidates to increase intestinal permeability [72]. The differences observed in terms of virulence between *E*. *histolytica* and *E*. *dispar* were indeed demonstrated to be due to the secretion of specific proteases and, specifically, to increased protease activity by *E*. *histolytica* [71].

In some cases, invasive stage highlights may contribute in providing new diagnostic tools. *Taenia solium* metacestodes are able to maintain a host infection by developing many protective mechanisms, including the production of ESPs. Such ESPs were investigated by Victor et al. [73] in an effort to unveil more proteins involved in parasite survival strategies as well as to better understand the interaction between metacestodes and their host.

ESP proteomic analysis was also performed in *A*. *caninum* [74], a model for human hookworm infections, providing insight into the biology of hookworm and immunomodulatory mechanisms by which these worms establish and maintain chronic infections in their host. The identified ESPs could be useful in the development of both anti-helminthic vaccines/drugs and therapeutic agents for inflammatory or autoimmune diseases. For example, 1 of these identified secreted proteins of *A*. *caninum*, the tissue inhibitor of metalloproteinase (TIMP)-like anti-inflammatory protein-2 (AIP-2), has been demonstrated to promote positive regulatory T-cell response and suppress airway inflammation in a mouse model, thereby showing a novel potential therapeutic drug for allergic asthma [75]. Also of interest is a recent proteomic work that investigated the physiological and biological influence of the gut microbiota on the parasite *T*. *spiralis*, suggesting that specific gut microbes may be considered as therapeutic agents to prevent trichinellosis [76].

### Host–parasite relationships and immune response

The host immune response and, consequently, the development and manifestation of chronic human inflammatory diseases may be modulated by infection with helminthic parasites, as in the case of *Trichuris trichiura*, which exerts a protective effect against atopy, and allergic and autoimmune diseases [77, 78]. Therefore, the immunomodulatory effect of *T*. *trichiura* adult worm extract was investigated to identify proteins acting as drug molecules for allergic and other inflammatory diseases [77]. The same authors characterised the adult stage transcriptome of *T*. *trichiura* [78], which contributed to the functional annotation of a recently released genome draft [79]. It is worthwhile to also mention the protein array technique (miniaturisation of thousands of assays on 1 small plate) facilitating the analysis of host immune response to parasite antigens; Tang and colleagues probed the serum of patients infected with *N*. *americanus* with an array of 564 recombinant proteins inferred from the genome of the parasite and identified 22 antigens that were significant targets of anti-hookworm immune responses and might form the basis of sensitive and specific serodiagnostic tests [49].

### Future direction for protein-based approach: New frontiers of post-translational modifications and protein–protein interactions

Until now, human gut parasite transcriptomics and proteomics have not been analysed in depth; modern technological MS-based platforms are able to perform more sophisticated analyses, such as the study of the intact protein complexes and the detection of direct protein–protein interactions, the application of which in the interpretation of host–parasite interaction networks will be of invaluable help in the battle against infection [80]. Elucidation of protein post-translational modifications (PTMs; i.e., phosphorylation, acetylation, glycosylation, etc.) is another fundamental target in research on parasites for their critical role in protein function and therefore in the progression and outcome of infection. As an example, histone PTMs of *E*. *histolytica* and *G*. *lamblia* might be involved in host–parasite interactions in terms of virulence and morphological differentiation [81].

## Metabolomics

The GI tract is a dynamic metabolic and immunologically active ecosystem, and its complete set of metabolites reflects both the enzymatic pathways of host and gut inhabitants and the complex network that connects them. Metabolomics aims to monitor metabolite components in a system and determine their quantitative dynamic change. Two technologies commonly associated with metabolome analyses are nuclear magnetic resonance (NMR) spectroscopy and gas or liquid MS, well suited for identification and quantitation of small-molecular-weight metabolites in a high-throughput fashion [82]. Recent findings describe the roles of microbial metabolites in regulating host physiology, immunity, and pathology [83–85]. Therefore, metabolomics provides a novel approach to studying the microbiota but also the “parasitome” and its interactions with the host counterpart. Indeed, perturbations of gut metabolite profiles reflect changes in cellular regulation and physiological processes that may result from parasitic infections, and these profiles may provide a pathway for biomarker discovery, drug targets, and improved diagnoses [23].

### From metabolomic patterns to diagnostic and therapeutic tools for protozoa

Despite the differences in faecal metabolite profiles in *Cryptosporidium*-infected humans [86] and mice [87], metabolomics clearly differentiates between infected and uninfected states. Such metabolic differential patterns may be useful for the diagnosis of *Cryptosporidium* infections and to improve microscopy or PCR-based diagnoses, which are often hampered by sensitivity limits due to low numbers of oocysts in faeces because of intermittent shedding. *Giardia* lacks mitochondria and depends on fermentative metabolism, showing unique metabolic pathways. Volatile organic compounds (VOCs) may therefore represent specific markers of *Giardia* infection in stools, hence presenting a potential role for the diagnosis of giardiasis [88].

Because the elucidation of the encystation process could further the improvement of control measures against parasitic infectious diseases, metabolic and transcriptomic changes occurring during the encystation of *E*. *invadens*, a relative of *E*. *histolytica* that infects reptiles, have been investigated [89]. The encystation process leads to decreased levels of most metabolites involved in glycolysis and of all nucleotides, while the intermediates of chitin biosynthesis, some biogenic amines, and γ-aminobutyric acid increase. Because chitin does not occur in vertebrates, its synthetic pathway represents an excellent parasite-specific target for developing new chemotherapeutics.

### Metabolomic patterns to unveil bacteria, parasite, and host interplay

VOC-based analysis, coupled with metagenomic analysis, as previously described, was performed on the luminal contents of pigs infected with *T*. *suis* [43]. Twenty-six percent of all identified colonic metabolic pathways were affected by *T*. *suis* presence, with a drop out of cofactors for carbohydrate and lysine biosynthesis. Moreover, the observed accumulation of oleic acid in IPs suggested altered fatty acid absorption, hence enhancing local inflammation. Therefore, *T*. *suis* exhibited a central role in the microbiota–host axis [43].

Wang and collaborators [90] pioneered the strategy of metabolic profiling of blood and urine to investigate biochemical consequences of *N*. *americanus* infection in an animal model to determine the host metabolic response. One of the prominent changes noted was the alteration of host energy-related metabolism, which was reflected in an increased concentration of lipoprotein and lipids and a decreased concentration of glucose in the blood. Additionally, a number of urinary metabolites was found to increase in infected hamsters, including *p*-cresol-glucuronide and 2-aminoadipate [90]. The same authors performed the same metabolic investigation after coinfection with *S*. *japonicum* and *N*. *americanus*, noting again a reduced concentration of the gut microbial-related metabolite hippurate in the hamster urine [91]. The decrease in hippurate levels is common to all helminth infections studied to date. However, it is evident that no single metabolite can be a specific marker for parasitosis: the metabolic signature itself, at least in theory, could be a diagnostic reference.

## Conclusions and perspectives

The strength of “omic” approaches resides in their ability to provide complete profiles of genes/transcripts/proteins/metabolites, overcoming the classical genetic/biochemical studies based on single or few target molecules, giving a broader perspective of parasite biology and, as a consequence, improving parasite control programs and diagnostics.

After reviewing studies based on “omic” technologies, possible markers of parasite infection have emerged, as well as putative vaccine targets. However, although interesting data have been revealed, considerable work remains to improve current “omics”-based operational pipelines in order to fully understand the 3-way interactions between host, prokaryotic communities, and parasites. Future research should include the extended sequencing of parasite genomes; one of the actual shortcomings of “omic” studies is the poor characterisation of new parasite genes both in their sequences and functions. More deeply curated annotations and affordable metagenomic pipelines are needed for the description of parasites within the gut microbiota environment. Moreover, new metaproteomic procedures (MS differential profiling by multiple reaction monitoring-like acquisition), now available for prokaryotic communities, need to be applied to eukaryotic citizens (the “parasitome”) of the gut ecological system in order to greatly improve the distinction between host and parasite proteins as well as to identify as-yet unknown proteins.

**Fig 2:**
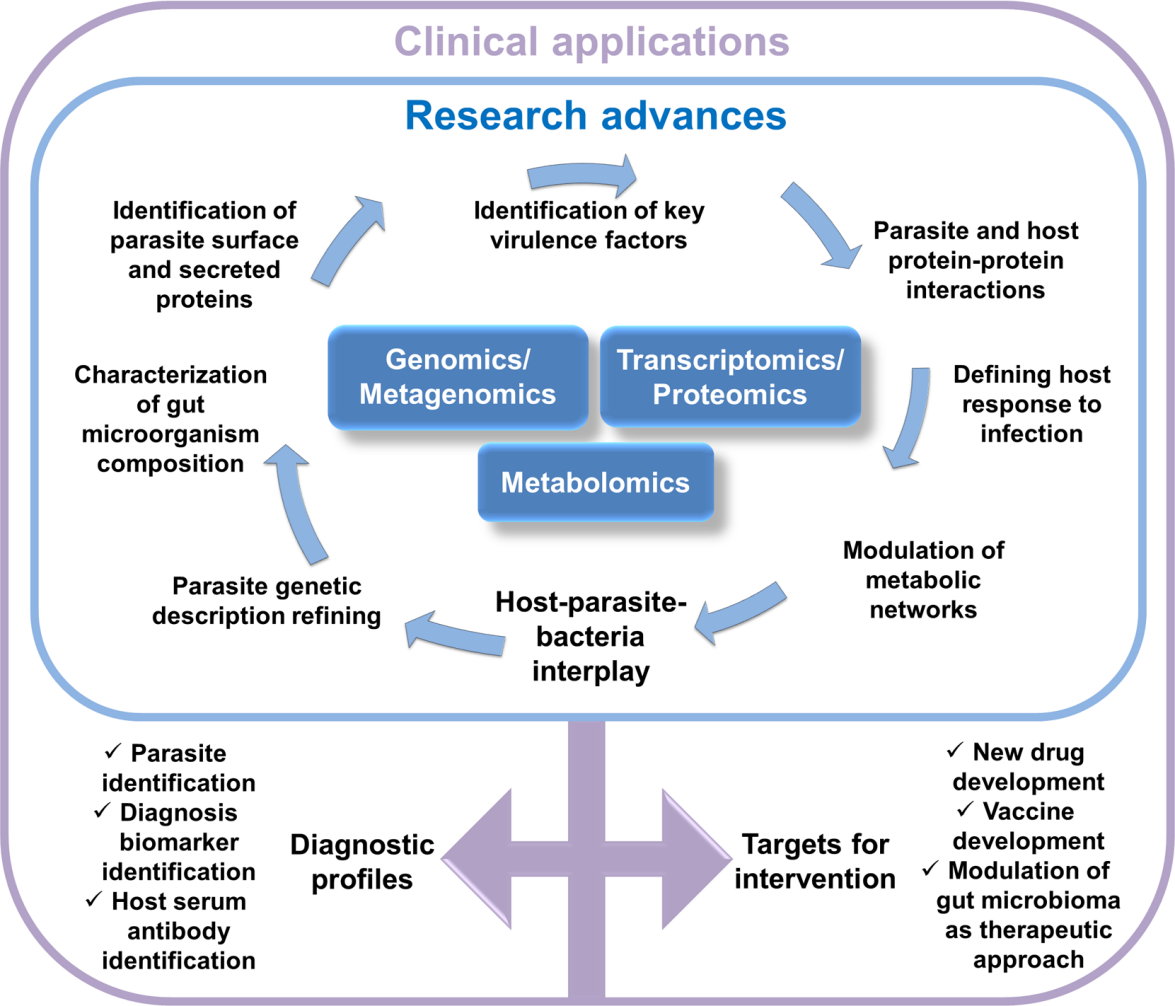
Multi-omic basic research will define molecular mechanisms on the basis of host–parasite–bacteria cross-talk on the road to more effective translational research.

Therefore, “omic” technologies are now promising tools capable of leading to the discovery of new key pathways which may improve diagnostic and therapeutic approaches for parasite-linked GI diseases within the context of microbiota/parasitome/host co-metabolism and response to infections (Fig 2).

**Key learning points**

- The human gut is a complex ecological system composed of host and symbiotic prokaryotic cells and plays a central role in human health. Intestinal eukaryotic parasites (i.e., protozoa and helminths) are the other important components of this coevolved community, being able to modify the composition/activity of the gut prokaryotes through excreted/secreted molecules or evoking a response from the host’s immune system. At the same time, bacteria may exert profound effects on parasite physiology and survival within the intestinal niche.
- The “omic” approaches (genomics, transcriptomics, proteomics, and metabolomics), based on the recent development of efficient analytical and data mining methods, allow the overall evaluation of gene/transcripts/protein/metabolite scaffolds of a biological system under specific conditions and time points. This offers non-targeted, high-throughput, and deep systems biology analyses that may be the key to decoding the functional activity of the human gut community.
- Metagenomic data pipelines, developed for studying prokaryotes, are valuable tools for the detection of eukaryotic DNA signatures in gut microbiota communities; existing metagenomic data from studies across geographical reservoirs can be used to produce standard profiles of prokaryotes of healthy populations, enabling us to identify dysbiosis. Such reference microbiota can be exploited to assess actual prokaryotic–eukaryotic relationships within the ecology of the human intestinal niche.
- A deeper “omic” and new “meta-omic” profiling of both the parasite and parasite–microbiota–host interplay will further assist the discovery of the entire biological machinery of the gut community and will have a valuable impact in unveiling new diagnostic and virulence markers as well as promising targets for vaccination.

**Top 5 papers**

1. Lee SC, Tang MS, Lim YA, Choy SH, Kurtz ZD, Cox LM, et al. Helminth colonization is associated with increased diversity of the gut microbiota. PLoS Negl Trop Dis. 2014;8(5): e2880.
2. Tang YT, Gao X, Rosa BA, Abubucker S, Hallsworth-Pepin K, Martin J, et al. Genome of the human hookworm *Necator americanus*. Nat Genet. 2014;46(3):261–9.
3. Biller L, Matthiesen J, Kuhne V, Lotter H, Handal G, Nozaki T, et al. The cell surface proteome of *Entamoeba histolytica*. Molecular & cellular proteomics: MCP. 2014;13(1):132–44.
4. Jiang HY, Zhao N, Zhang QL, Gao JM, Liu LL, Wu TF, et al. Intestinal microbes influence the survival, reproduction and protein profile of *Trichinella spiralis* in vitro. International journal for parasitology. 2016;46(1):51–8.
5. Bond A, Vernon A, Reade S, Mayor A, Minetti C, Wastling J, et al. Investigation of Volatile Organic Compounds Emitted from Faeces for the Diagnosis of Giardiasis. J Gastrointestin Liver Dis. 2015;24(3):281–6.
